# The development of self-regulated learning during the pre-clinical stage of medical school: a comparison between a lecture-based and a problem-based curriculum

**DOI:** 10.1007/s10459-015-9613-1

**Published:** 2015-05-29

**Authors:** Susanna M. Lucieer, Jos N. van der Geest, Silvana M. Elói-Santos, Rosa M. Delbone de Faria, Laura Jonker, Chris Visscher, Remy M. J. P. Rikers, Axel P. N. Themmen

**Affiliations:** Institute of Medical Education Research Rotterdam, Erasmus MC, Room AE-239, PO Box 2040, 3000 CA Rotterdam, The Netherlands; Department of Neuroscience, Erasmus MC, Rotterdam, The Netherlands; Department of Medicine, Federal University of Minas Gerais, Belo Horizonte, Brazil; Department of Medicine, Unifenas – Universidade José do Rosária Vellano, Alfenas, Brazil; Center for Human Movement Sciences, University Medical Center Groningen, University of Groningen, Groningen, The Netherlands; Department of Psychology, Erasmus University Rotterdam, Rotterdam, The Netherlands; Department of Internal Medicine, Erasmus MC, Rotterdam, The Netherlands; Department of Sociel Sciences, Utrecht University, University College Roosevelt, Middelburg, The Netherlands

**Keywords:** Lectured-based instruction, Lifelong learning, Medical education, Problem-based learning, Self-regulated learning

## Abstract

Society expects physicians to always improve their competencies and to be up to date with developments in their field. Therefore, an important aim of medical schools is to educate future medical doctors to become self-regulated, lifelong learners. However, it is unclear if medical students become better self-regulated learners during the pre-clinical stage of medical school, and whether students develop self-regulated learning skills differently, dependent on the educational approach of their medical school. In a cross-sectional design, we investigated the development of 384 medical students’ self-regulated learning skills with the use of the Self-Regulation of Learning Self-Report Scale. Next, we compared this development in students who enrolled in two distinct medical curricula: a problem-based curriculum and a lectured-based curriculum. Analysis showed that more skills decreased than increased during the pre-clinical stage of medical school, and that the difference between the curricula was mainly caused by a decrease in the skill evaluation in the lecture-based curriculum. These findings seem to suggest that, irrespective of the curriculum, self-regulated learning skills do not develop during medical school.

## Introduction

It is widely recognized that medical doctors are expected to stay updated with developments in their field (Greveson and Spencer 2005; Brydges and Butler 2012) and maintain and improve their competencies (Artino et al. 2012; Brydges and Butler 2012; Dannefer and Prayson 2013). To be a lifelong learner, it is important to manage one’s learning by actively taking control of one’s learning activities (Ertmer and Newby 1996; Lycke et al. 2006; Brydges et al. 2012), also known as self-regulated learning (Zimmerman 1989; Wolters 1998). According to Ertmer and Newby (1996), self-regulated learners are individuals who plan and organize their learning activities, set goals, monitor their progress, reflect upon, and evaluate their learning process.

Hong and O’Neill (2001) added two motivational components that play an important role in self-regulated learning: effort and self-efficacy. Self-regulated learners show high levels of effort, they persist and reach the goals that they have set. Hong and O’Neill also consider self-efficacy as a key factor in the learning process, since it refers to the amount of trust someone has in his/her own potential to complete the task. Learners need to believe in their own abilities to successfully complete a task and to be motivated to use self-regulated learning skills (Hong and O’Neil 2001; Sungur and Tekkaya 2006). Since the need for highly self-regulated, lifelong learning medical doctors is widely recognized, medical education is based upon the premise that students should be encouraged to develop self-regulated learning skills, since this will enable them to continue learning in their professional career (Greveson and Spencer 2005).

Curricula can take different forms, based on the educational approach that is used for the students’ development. One approach with a long-standing history is the teacher-centered, lectured-based (LB) curriculum. Lectures are known to be an effective method to transfer a large quantity of information from instructor to student (Liker et al. 1990). One of the prevailing alternatives to an LB-curriculum is a problem-based learning (PBL) curriculum. Although variation in implementation of PBL exists, all implementations share core characteristics. That is, the education is student-centered, students study in small groups that are guided by a tutor, a problem is discussed to start the learning process and activate students’ prior knowledge, and after the discussion, students spent considerable time on self-study while only a few complementary lectures are provided (Evensen et al. 2001; Barrows 2002; Dolmans and Wolfhagen 2010; Wijnia et al. 2011). PBL-curricula thereby emphasize self-regulated learning and thus lifelong learning skills (Dolmans and Schmidt 1994; Lloyd-Jones and Hak 2004; Loyens et al. 2006). Several studies have investigated the development of self-regulated learning skills in PBL-settings, in preclinical and clinical medical education but also in other disciplines, and concluded that PBL students become better self-regulated learners during their education, due to the active participation and discussions required in the PBL-curriculum (Vernon and Blake 1993; Dolmans and Schmidt 1994; Lycke et al. 2006; Downing et al. 2009; Wijnia et al. 2011). However, it has also been argued that all students will adopt some self-regulated learning skills, independent of the educational approach (Loyens et al. 2008).

The present study was devised to answer two questions. First, do self-regulated learning skills develop in the pre-clinical phase of medical school irrespective of the curriculum? We hypothesize that it does, since medical schools aim to graduate medical doctors who are able to be lifelong learners (Greveson and Spencer 2005). Second, do self-regulated learning skills develop more in students enrolled in the pre-clinical stage of a PBL-curriculum than in those the pre-clinical stage of a LB-curriculum? Since previous studies reported contradictory findings (Vernon and Blake 1993; Dolmans and Schmidt 1994; Lycke et al. 2006; Loyens et al. 2008) it is interesting to investigate this assumption, as it would indicate that the development of self-regulated learning is better supported in PBL. Our hypothesis is that students in a PBL-curriculum will show a stronger development in self-regulated learning skills during the pre-clinical stage of medical school, given that these skills are more explicitly incorporated in a PBL-curriculum than in a LB-curriculum (Lloyd-Jones and Hak 2004; Loyens et al. 2006) as PBL focuses on active engagement of students in their learning process (Dolmans and Schmidt 1994; Evensen et al. 2001).

## Methods

### Setting

This quasi-experimental cross-sectional study took place at two different medical schools in Belo Horizonte, Brazil. The first medical school selected for this study was a medical school where education is lectured-based. The second medical school selected was one where a PBL approach has been implemented 9 years ago. At both medical schools, the total duration to obtain the MD degree was 12 semesters (6 years).

The students admitted are of a comparable level, since medicine is a very competitive field in Brazil and only those with very high qualifications are accepted to enter medical school (Castro 2012). In addition, both medical schools have been recently evaluated by the governmental institution in charge of accreditation of Brazilian medical school [The Brazilian National Institute for Educational Studies and Research-Ministry of Education (INEP-MEC)]. The evaluation scores are based, among others, on on-site evaluations of the courses, the quality of courses, the structure of the institution, and the students grades on a national academic examination. Both medical schools received an average grade of 4 out of 5 (http://emec.mec.gov.br).

### Participants

Four different groups were included in this study: the second and sixth semester students of the lectured-based medical school (LB-2 and LB-6) and of the problem-based medical school (PBL-2 and PBL-6). The second and sixth semester students were chosen for this study to reduce the effect of pre-university influences and to maximize the possible influence of the medical school itself on the development of self-regulated learning during the pre-clinical stages of medical education. In total, 478 students were approached to participate in this research, 384 completed the questionnaire, giving an overall response rate of 80.3 %. The mean age of all respondents was 22.4 years (*SD* 4.0 years, range 18–46); 59.9 % was female. The response rate in LB-2 was 70.7 % (111/157), in this group, the mean age was 20.6 years (*SD* 3.3, range 18–36) and 54 % of the respondents was female. In LB-6, the response rate was 82.7 % (139/168), the mean age was 23.6 (*SD* 3.6, range 19–40) and 60.1 % of the respondents was female. In PBL-2, the response rate was 85.7 % (66/77), the mean age was 21.7 (*SD* 4.6, range 18–46) and 63.6 % of the respondents was female and in PBL-6, the response rate was 89.5 % (68/76) and in here, the mean age was 23.5 (*SD* 4.3, range 20–43) and 63.2 % of the respondents was female. The groups do not significantly differ in percentage of females, and the second semester and sixth semester students are comparable in age, respectively. The lower number of students from the PBL-curriculum is the result of the lower number of students enrolled in this medical school.

### Instrument

#### Questionnaire: Self-Regulation of Learning-Self-Report Scale

The students’ level of self-regulated learning skills was investigated using the Self-Regulation of Learning-Self-Report scale (SRL-SRS), which contains 50 items, each with a 4 or 5-point Likert scale, depending on the subsection of the questionnaire. The subscales are planning, monitoring, evaluation, reflection, effort, and self-efficacy, based on the definitions of self-regulated learning stated by Ertmer and Newby (1996), and Hong and O’Neill (2001). For example, a question in the subscale monitoring is: “While making an assignment, I check my progress,” and an example from the subscale effort is: “I keep trying to finish my assignment, even when I find the assignment extremely difficult”. The questionnaire has been composed and validated in a Dutch study. In this study, two confirmatory factor analyses were conducted involving 601 and 600 adolescents aged 11–17 years to test and cross-validate the six-factor model. These analyses, plus a relative and an absolute test–retest reliability showed the questionnaire to be a valid and reliable measure of self-regulated learning (Toering et al. 2012). The guidelines described by Beaton et al. (2000) were used to translate the questionnaire to Brazilian–Portuguese.

### Procedure

Within a 5-week period, all second and sixth semester students of both universities were asked to voluntary participate in the study. The students were informed that the questionnaire was aimed to gain more insight in their study behavior. The students provided their email address to receive a link of the online version of the questionnaire that they could immediately complete in a computer room, or they received the questionnaire on paper in their classroom. The questionnaire took approximately 20 min to complete and a small gift was provided after completion.

### Data analysis

The data were analyzed with IBM SPSS AMOS 20 and IBM Statistics SPSS 20. confirmatory factor analysis (CFA) was used to investigate whether the constructs of the questionnaire fitted still the model, since the questionnaire was translated and completed by students instead of adolescents. Cronbach’s alphas were calculated to measure the internal consistency of the factors.

Analysis of variance (ANOVA) was to address the first question, to compare the level of self-regulated learning skills between the second semester students and sixth semester students and to analyze the overall development of self-regulated learning skills. A *p* value of <.05 was considered significant. In addition, effect sizes, eta squared (*η*^2^), were converted. Values of .01, .06 and .14 indicate small, medium or large effects, respectively (Cohen 1988; Lakens 2013).

For the second question, whether there was a difference in development in SRL skills between the two curricula, the interaction effect between semester and curriculum with an ANOVA general linear model was analyzed. Here, also a *p* value of <.05 was considered significant and *η*^2^ were converted as well.

In order to gain more understanding about the results of these analyses, the difference between the second semester students was analyzed to determine which of the baseline measures differed, and the differences within the LB-curriculum and within the PBL-curriculum were assessed with ANOVA. Again, a *p* value of <.05 was considered significant and eta squared (*η*^2^) were converted.

### Ethics

Ethical approval for this study in both medical schools was received from the ethical committee COEP/UFMG (case number CAAE—0469.1.203.203-11). Students were informed about the study and had the choice to participate following the rules of informed consent.

## Results

### Validation of the questionnaire

To determine the goodness of fit of the model, a confirmatory factor analysis (CFI) was performed. Missing values (<1 % of the data) were replaced by mean scores (Ludbrook 2008). The original six-factor model showed a moderate fit; the CFI was .86 (Byrne 2010). The values of CMIN/d.f. and RSMEA were reasonable, since a CMIN/d.f. score below 3.0 is required and for the RMSEA a score <.06 (Byrne 2010). In the original model, factor loadings of one item of the subscale planning, one of effort and two of the subscale self-efficacy were low. By removing these items in this order, we came to an adjusted model that showed a good fit; a CFI of .902, a CMIN/d.f. of 1.8 and a RSMEA of .045. Table 1 provides summary of the χ^2^ values and χ^2^ differences of the adjusted model described above compared to a model where all factor loading constrained equal. The χ^2^ difference between these two models was not significant indicating that the same constructs were measured across the four student groups in the present study: the second and sixth semester students of the LB curriculum and the second and sixth semester students of the PBL curriculum. Therefore, the adjusted model was chosen to analyze the data. The internal consistency of the adjusted factors was strong, both for the entire group as for all four separate groups (see Table 2) and did not improve noteworthy when any other item within the factors was removed.

**Table 1:** The model summary of goodness of fit statistics for tests for invariance indicated that the same constructs were measured across the four student groups

Model description	χ^2^	Df	Δχ^2^	ΔDf	Statistical significance
Adjusted model	1435.547	804			
Model with factor loadings constrained equal	4889.067	3216	3453.480	2408	NS

*χ* ^2^, Chi square; Δχ^2^, difference in Chi square between the models; ΔDf, difference in number of degrees of freedom between the two models; NS, not significant at .05 level

**Table 2:** Descriptive statistics and alpha reliabilities for the adjusted six factors of the Self-Regulated Learning Self Report Scale

Factor	No of items	Minimum	Maximum	Mean	Alpha reliability entire group	Alpha reliability LB2	Alpha reliability LB6	Alpha reliability PBL2	Alpha reliability PBL6
Planning	7	8	28	21.3	.84	.85	.82	.80	.82
Monitoring	6	8	24	19.2	.83	.85	.82	.80	.82
Evaluation	8	17	40	33.0	.84	.87	.89	.89	.88
Reflection	5	13	25	22.5	.85	.83	.75	.80	.79
Effort	8	11	32	24.6	.83	.83	.81	.82	.86
Self-efficacy	8	13	32	23.8	.80	.82	.82	.72	.82

### Comparison of factor scores between and within universities and semesters

Table 3 shows the results of the comparisons of the level of self-regulated learning skills between all second and sixth semester students, irrespective of their curriculum. The analysis shows that three of the six self-regulated learning skills developed negatively between the second and sixth semester of medical school. This decrease is seen for the skills planning; *F*(1383) = 4.743, *p* = .030, *η*^*2*^ = .012; monitoring; *F*(1383) = 7.987, *p* = .005, *η*^*2*^ = .021; and evaluation; *F*(1383) = 9.250, *p* = .003, *η*^*2*^ = .024, although the effect sizes are small. The level of the other self-regulated learning skills, reflection, effort and self-efficacy, did not change between the second and sixth semester.

**Table 3:** The self-regulated learning skills planning, monitoring and evaluation slightly decrease between the second semester and sixth semester

	N	Planning	Monitoring	Evaluation	Reflection	Effort	Self-efficacy
Mean semester 2	177	21.7	19.7	33.8	22.3	25.0	23.6
Mean semester 6	207	20.9	18.8	32.4	22.7	24.3	23.9
Test value (*F*)		4.743	7.987	9.250	2.196	3.236	.434
*p* value		.030	.005	.003	.139	.073	.510
*η* ^*2*^		.012	.021	.024			

*η* ^2^ = effect size, where .01, .06 and .14 indicate small, medium and large effect

A *p* value <.05 is considered significant

### Differences in development of self-regulated learning skills between curricula

To determine the difference in development of self-regulated learning skills between the different curricula, the interaction effect of semester and curriculum on each of the six subscales was assessed with an ANOVA. The results are displayed in Table 4. The only, large, significant interaction effect was found on the skill evaluation, *F*(1382) = 6.718, *p* = .010, *η*^*2*^ = .256.

**Table 4:** The self-regulated learning skill evaluation develops differently in the two curricula

	Planning	Monitoring	Evaluation	Reflection	Effort	Self-efficacy
Test value (*F*)	.399	2.118	6.718	3.350	1.849	1.179
*p* value	.528	.146	.010	.068	.175	.278
*η* ^*2*^			.256			

*η* ^2^ = effect size, where .01, .06 and .14 indicate small, medium and large effects

A *p* value <.05 is considered significant

Analyses of the difference between second semester students of the LB-curriculum and the PBL-curriculum, as well as of the difference between the second and sixth semester students within each curriculum, show that this effect is caused by a decrease in the subscale evaluation in the LB curriculum *F(*1249) = 15.506, *p* < 001, *η*^*2*^ = .059 (see Table 5).

**Table 5:** The difference in the development of the skill evaluation between the curricula is caused by a negative development of this skill in the LB-curriculum

	N	Planning	Monitoring	Evaluation	Reflection	Effort	Self-efficacy
Mean LB 2	111	21.6	19.7	33.7	22.3	24.2	23.5
Mean PBL 2	66	22.0	19.8	33.9	22.4	26.3	23.8
Test value (*F*)		.439	.029	.085	.053	12.112	.200
*p* value		.509	.865	.771	.819	.001	.656
*η* ^*2*^						.065	
Mean LB 2	111	21.6	19.7	33.7	22.3	24.2	23.5
Mean LB 6	139	20.6	18.5	31.5	22.4	23.9	23.5
Test value (*F*)		4.415	9.664	15.506	.039	.285	.002
*p* value		.037	.002	<.001	.845	.594	.964
*η* ^*2*^		.018	.038	.059			
Mean PBL 2	66	22.0	19.8	33.9	22.4	26.3	23.8
Mean PBL 6	68	21.5	19.5	34.1	23.3	24.9	24.7
Test value (*F*)		.516	.205	.111	6.529	4.272	1.883
*p*-value		.474	.651	.740	.012	.041	.172
*η* ^*2*^					.047	.031	

*η* ^2^ = effect size, where .01, .06 and .14 indicate small, medium and large effects, a *p* value <.05 is considered significant

## Discussion

The first aim of the present study was to investigate the development of medical students’ self-regulated learning skills during the pre-clinical stage of medical school. Instead of the expected increase in skills, we found that three self-regulated learning skills, e.g. planning, monitoring and evaluation, slightly decreased between the second and sixth semesters, while other skills did not change. Previous research showed a positive development of students’ use of self-regulated learning skills within just 15 months of enrollment (Downing et al. 2009). We were therefore surprised to find no or even a negative development in 2 years. The scores on the subscale reflection were already quite high during the second semester and could therefore not improve much anymore, but the scores for other skills showed room for development during medical school.

Studies have suggested that the development of self-regulated learning skills can be distorted by various factors (Moust et al. 2005; Schmidt et al. 2011; Frambach et al. 2012). Students need to have time to learn how to self-regulate their learning (Bjork et al. 2013). Busy time schedules could obstruct this process since they severely constrain the amount of time students can invest in the development of self-regulated learning skills (Schmidt et al. 2011). It has also been shown that there is a negative correlation between the use of self-regulated learning skills and the dependence on a teacher or tutor; when students receive more structured education, they use less self-regulated learning skills (Premkumar et al. 2013). Finally, research has indicated that there are mixed findings on how accurate students’ reports of self-regulated learning are. That is, they tend to overestimate their self-regulated learning skills (Zimmerman 2008), and it might be the case that the second semester students overestimated their skills more than the sixth semester students, which could be a reason why we did not find a development in self-regulated learning skills. These issues require more attention in future research. Another suggestion for further research is to also include fourth semester students, in addition to second and sixth semester students, when investigating the development of self-regulated learning skills in medical education. The inclusion of fourth semester students would provide more accurate information on how self-regulated learning evolves during medical school, for instance, when does the negative development of some self-regulated learning skills begins?

For the second aim of this study, we compared the development of self-regulated learning skills between a LB and a PBL-curriculum. Here, we found a difference in the development of the skill evaluation between the two curricula. A closer look showed that this difference was caused by a negative development of evaluation in the LB-curriculum. Next to this, in the LB-curriculum, sixth semester students scored also lower on planning and monitoring than second semester students. In the PBL-curriculum, second semester students started with a relative high level of effort but this level drops a little. Interestingly, the level of reflection does develop slightly in the PBL-curriculum. Thus, although some skills developed negatively and the skill reflection increased a little in the PBL-curriculum, we found, unlike previous studies (Vernon and Blake 1993; Dolmans and Schmidt 2006; Lycke et al. 2006), only a significant difference in the development of the self-regulated learning skill evaluation between the curricula, and this difference is not caused by a positive development but by a negative development in the LB-curriculum. An explanation for the reason why the self-regulated learning skills did not develop more in the PBL-curriculum, may be that there are differences between the educational psychology principles of PBL and their implementation in a curriculum (Moust et al. 2005). In fact, there are some differences in definitions and implementations of PBL (Charlin et al. 1998; Ates and Eryilmaz 2010) and the implementation can even change over time within universities (Baroffio et al. 2013). As a result, emphasis on self-regulated learning can differ between PBL-curricula. In addition, the organization of the education, such as the quality of the learning material (Peterson 2004; Azer et al. 2013; Baroffio et al. 2013) and the role of the tutor, are crucial for the successful implementation of PBL (Peterson 2004; Ates and Eryilmaz 2010; Azer et al. 2013; Baroffio et al. 2013). PBL can also evoke feelings of uncertainty among students. They may respond to this uncertainty by relying more upon other students’ advice for planning and studying and consequently do not enough use and develop their own self-regulated learning skills (Baroffio et al. 2013).

Culture also seems to influence the effect of PBL (Frambach et al. 2012). Since it has been argued that self-regulated learning relies on Western principles, Frambach and colleagues investigated the cultural effect on the implementation of PBL in the Netherlands, Hong Kong, and Middle East (Frambach et al. 2012). They showed that students from the Middle East were more uncertain than students from the Netherlands and Hong Kong, and that these students felt lost in the PBL-system. In addition, a strong teacher-centered type of secondary education, as in Brazil, may hinder students’ development of self-regulated learning (Moust et al. 2005; Frambach et al. 2012). However, in Frambach’s study, all students appeared to have internalized the concept of self-regulated learning between the first and third year (Frambach et al. 2012), while we were not able to conclude this for the Brazilian students. Future research should therefore examine the quality of the implementation of PBL and the cultural effects.

One of the limitations of the present study is the use of a cross-sectional design, where a longitudinal design might have been more informative. However, since the groups are comparable in age and gender, the sample sizes are large, and response rates are comparable, we consider a cross-sectional design to be adequate. Another limitation is that we could not completely control for differences between the two universities. Although both medical school received the same evaluation score from The Brazilian National Institute for Educational Studies and Research-Ministry of education, it is possible that the pre-university learning skills of the student groups were different, since one university was publicly funded (LB) and the other was privately funded (PBL). However, this difference was most likely small. Medicine is one of the most competitive educational areas in Brazil with 150 applicants competing for one position. As a consequence, only those applicants with the highest scores on a national admission exam (ENEM/SISU; exam and scores are provided by the Brazilian Ministry of Education) are accepted for medical school (Castro 2012). In addition, except for the skill effort, the students’ self-regulated learning skills were comparable in the 2nd semester, which indicates that the level of self-regulated learning of the second semester students was comparable.

A third limitation is that some students completed the questionnaire on paper while others completed the questionnaire online. However, this limitation is relatively minor since the circumstances under which the questionnaire was completed were equal, they were all completed in a classroom at the medical school, and research indicated that paper and online versions of questionnaires can be taken as equivalent (Vallejo et al. 2007). Another limitation is that we investigated the development in self-regulated learning skills between second and sixth semester students, and not between first and sixth semester students. One could argue that the first semester already may have influenced the findings. The decision to include second semester students was deliberate, since we wanted the students to report on their experience in medical school when answering the questions, and not on the probably very intensive study behavior they must have shown to pass their entry exams. In addition, several studies have shown that many PBL students initially have difficulties in adjusting to the program. They lack confidence in active learning and rely heavily on their tutors, instead of defining their own learning goals (Schmidt et al. 1993; Miin et al. 1999; Lee et al. 2010). Furthermore, just the subscale effort and none of the other subscales were different between the second semester students. We thus do not expect that our findings are biased due to the inclusion of second semester students. A final limitation is that our group sizes are different. This is however a result of the difference in places available to study medicine in both universities and did most likely not influence the findings in this study.

## Conclusions

Although medical schools, and in particular those with a PBL-curriculum, are based upon the premise that their graduates should become self-regulated learners, our study shows that most self-regulated learning skills of medical students do not develop during the pre-clinical stage of medical school, while some skills even decrease. This can be a result of the lack of stimulation of self-regulated learning due to the structure of the pre-clinical curriculum. Especially in a PBL-setting, the quality of the implementation of the curriculum and the cultural background of the students may influence the development of self-regulated learning. In sum, medical schools should carefully consider the actions they take to improve the development of self-regulated learning skills in their students.
